# Associations between serotonin transporter gene polymorphisms and heat pain perception in adults with chronic pain

**DOI:** 10.1186/1471-2350-14-78

**Published:** 2013-07-30

**Authors:** W Michael Hooten, William R Hartman, John Logan Black, Heidi J Laures, Denise L Walker

**Affiliations:** 1 Department of Anesthesiology, Mayo Clinic College of Medicine, Rochester, MN 55905, USA; 2 Department of Laboratory Medicine and Pathology, Mayo Clinic College of Medicine, Rochester, MN 55902, USA; 3 Department of Anesthesiology Division of Pain Medicine, Mayo Clinic College of Medicine, 200 First St SW, Rochester, MN 55905, USA

## Abstract

**Background:**

The triallelic serotonin transporter gene linked polymorphic region (5-HTTLPR) has been associated with alterations in thermal pain perception. The primary aim of this study was to investigate the associations between heat pain (HP) perception and the triallelic 5-HTTLPR in a large cohort of adults with chronic pain.

**Methods:**

The cohort included 277 adults with chronic pain who met inclusion criteria, and were consecutively admitted to an outpatient pain rehabilitation program from March 2009 through March 2010. Individuals were genotyped for the triallelic 5-HTTLPR (including rs25531) and categorized as high, intermediate, or low expressors of the serotonin transporter. Standardized measures of HP perception were obtained using a validated quantitative sensory test method of levels.

**Results:**

The distribution of the high, intermediate, and low expressing genotypes was 61 (22%), 149 (54%) and 67 (24%), respectively. The Hardy-Weinberg *P*-value was 0.204 which indicated no departure from equilibrium. A significant effect of genotype was observed for values of HP threshold (*P* = 0.029). Individual group comparisons showed that values of HP threshold were significantly greater in the intermediate compared to the high expressing group (*P* = 0.009) but not the low expressing group (*P* > 0.1). In a multiple variable linear regression model, the intermediate group (*P* = 0.034) and male sex (*P* = 0.021) were associated with significantly greater values of HP 0.5, but no significant genotype-by-sex interaction effect was observed.

**Conclusions:**

In this study that involved adults with chronic pain, the intermediate triallelic 5-HTTLPR expressing group, but not the low expressing group, was associated with greater HP thresholds compared to the high expressing group.

## Background

The effects of serotonin (5-HT) on pain are influenced by the 5-HT transporter (5-HTT) which facilitates 5-HT reuptake from the synaptic cleft [1]. The gene coding for 5-HTT (*SLC6A4*) has a 43 base-pair insertion/deletion in the 5’- regulatory promoter region which is commonly referred to as the 5-HTT linked polymorphic region (5-HTTLPR) [2,3]. The 5-HTTLPR consists of a long (l) and a short (s) allele. The s-allele variant has been associated with reduced transcription efficiency with resultant reductions in 5-HTT expression and 5-HT reuptake. An A to G substitution (rs25531) in the 5-HTT promoter has also been described [4,5]. This single nucleotide polymorphism (SNP) has been found to influence 5-HTT gene expression such that the minor G-allele, which is nearly always in phase with the l-allele, reduces the efficiency of 5-HTT transcription to s-allele levels. These two polymorphisms, when studied jointly, have been referred to as the triallelic 5-HTTLPR, and individuals can be categorized as high (L_A_/L_A_), intermediate (S_A_/L_A_, L_A_/L_G_), or low expressors (S_A_/S_A_, L_G_/S_A_) of 5-HTT. Previous investigators have suggested that 5-HTTLPR polymorphisms may account for a two-fold difference in mRNA levels [6,7].

The 5-HTT has important effects on thermal pain perception in preclinical studies [8,9]. In human studies, 5-HTTLPR polymorphisms have been associated with differences in thermal pain perception in some, but not all, investigations that have predominately involved healthy volunteers [10-14]. However, the associations between heat pain perception and the triallelic 5-HTTLPR polymorphism have not been reported for a large cohort of adults with chronic pain. This is of particular importance because individuals with chronic pain have alterations in thermal perception [15-17], and the previously reported associations between thermal pain perception and the triallelic 5-HTTLPR polymorphism may be varied among adults with chronic pain. Thus, the primary aim of this study was to investigate the associations between heat pain (HP) perception and the triallelic 5-HTTLPR polymorphism in a cohort of adults with chronic pain.

## Methods

### Participants

The study protocol was approved by the Mayo Foundation Institutional Review Board, and written informed consent was provided by all patients prior to study participation. All adults consecutively admitted to the Mayo Comprehensive Pain Rehabilitation Center from March 2009 to March 2010 were eligible for study recruitment. During this time period, 524 patients were admitted, and 300 met inclusion criteria and were successfully recruited for study participation. Inclusion criteria included admission to the outpatient pain treatment program, chronic non-cancer pain greater than 3 months duration, and age ≥ 18 years. Exclusion criteria included the presence of a major medical (e.g., severe cardiac or pulmonary disease), surgical (e.g., spine or intraabdominal surgery within 6 months of admission) or psychiatric disorder (e.g., schizophrenia, dementia) that precluded full participation in the 3-week outpatient treatment program.

The genotype of one subject could not be determined, and baseline quantitative sensory test (QST) results were not obtained on 22 subjects due to lack of available study personnel. The distribution of high, intermediate and low expressing genotypes among the 22 subjects who did not have baseline sensory testing was 6 (27%), 9 (41%) and 7 (32%), respectively. Thus, the study cohort was comprised of 277 subjects for whom both genotype and QST data were available.

### Study setting

The outpatient pain rehabilitation program was of 3-weeks duration, and patients attended 8-hours daily. The clinical setting has been previously described [18]. To briefly summarize, a cognitive behavioral model served as the basis for treatment, and the primary goal of the outpatient program was restoration of physical and emotional functioning. The majority of patients have previously used multiple trials of analgesic medications, undergone surgeries, and utilized various interventional pain procedures with incomplete pain relief. Examples of common admitting diagnoses include low back pain, fibromyalgia and chronic headache. Patients were involved in daily physical and occupational therapy, and all patients attended daily educational group sessions related to management of depressive symptoms, relaxation training, stress management, activity moderation, and elimination of pain behaviors.

### Demographic and clinical characteristics

Baseline demographic and clinical characteristics were collected at admission including age, sex, pain duration, marital status, years of education, employment status, primary pain site, and smoking status.

### Determination of morphine equivalent dose

Upon admission, the daily opioid dose of each patient was determined by self-report and review of pharmacy records, as previously described [19]. The daily opioid dose was converted to daily morphine equivalents using an equianalgesic conversion software program [20] that has been used at our outpatient treatment center [19,21,22].

### Measures

#### ***Pain severity***

Pain severity was assessed using the pain severity subscale of the Multidimensional Pain Inventory [23]. This self-report questionnaire has proven reliability and construct validity [24]. The pain severity subscale is quantified from the responses to the following three questions: 1) “Rate the level of your pain at the present moment”; 2) “On the average, how severe has your pain been during the last week”; and 3) “How much suffering do you experience because of your pain.” Raw scores were converted to standardized *t*-scores with a normative value of 50 (range 0–100) and a SD of 10 [25].

#### ***Heat pain perception***

Heat pain perception was assessed using the automated Computer Aided Sensory Evaluator IV (CASE IV; WR Electronics, Stillwater, MN) system based on the method of levels [26-28], as previously described [16]. The CASE IV system delivers discrete magnitudes of heat stimuli, in units termed “just noticeable difference” (JND), interspersed with null stimuli in random order through a thermode with a surface area of 10 cm^2^. The baseline temperature was 34°C, and the thermal rise rate was 4°C per second. The levels of stimuli increased exponentially and achieved a maximal peak at 48°C at level 21. For levels 22, 23 and 24, the temperature of the thermode was raised to 48°C for a duration of 1.5, 5 and 10 seconds, respectively. At level 25, which was the maximal heat stimulus, the temperature was raised to 49°C for a 10 second duration. The subject was masked to the magnitude of the heat stimulus and whether or not a null stimulus was delivered. After each stimulus, including all null stimuli, the subject was asked to grade the intensity of the stimulus on an 11-point rating scale where 0 denoted no pain and 10 signified the most possible intense pain. The test was completed when either the subject rated the intensity of a heat stimulus ≥ 5 or when the maximum stimulus had been delivered, which avoids tissue injury. A quadratic regression equation was then fitted to the pain ratings from which HP 0.5, HP 5 and HP 5–0.5 were calculated by the CASE IV software program (WR TestWorks, version 2.0) [29,30]. Heat pain 0.5 was the midpoint between a nonpainful stimulus and the least stimulus magnitude necessary to elicit a threshold sensation of pain (Additional file 1: Figure S1). Heat pain 5 was the stimulus magnitude necessary to elicit a pain rating of 5 from the subject. It is important to note that HP 5 was a measure of intermediately intense pain, and not a measure of maximal heat pain tolerance. Heat pain 5–0.5 was the difference between HP 5 and HP 0.5, and has been termed the pain-stimulus response slope. The standardized test procedures, stimulus waveform, null stimulus, and the non-repeating stepping algorithm between the different levels of heat stimuli have been validated, and the HP test algorithm requires less than 5 minutes to complete [26-28,31].

Quantitative sensory testing was performed in a specifically designated room. All patients using opioids were on stable dosages at the time of testing. All study participants were tested using the dorsum of the nondominant hand. This particular anatomical site was chosen because of easy accessibility which was an important feasibility consideration with regards to conducting QST in an ambulatory care setting.

### Genotyping

DNA was extracted from whole blood at the Biospecimens Accessioning Processing Laboratory at Mayo Clinic Rochester using an automated platform (AutoGen FlexStar Qiagen Chemistries), quantification was by UV absorbance and quality was accessed by 260/280 OD ratio. The simultaneous determination of the long and short form of the 5-HTT promoter region and rs25531 was performed by PCR amplification of the promoter region of 5-HTT using primers 5’-TCCTCCGCTTTGGCGCCTCTTCC and 5’-TGGGGGTTGCAGGGGAGATCCTG, followed by Hpa II digestion of the resulting amplicon as described by Wendlend et al. [5,32]. Samples were denatured for 1 cycle of 2 min at 94°C, annealed for 30 cycles at 94°C for 30 sec, 58°C for 30 sec, 72°C for 1 min, and elongated for 10 min at 72°C. Digested fragment sizes of 512 and 469 bp correspond to L_A_ and S_A_ (long or short allele with “A” present at rs25531), respectively. The presence of G at rs25531 is indicative of a digested fragment of 402 bp, and an additional fragment of 69 or 100 bp for the short and long allele, respectively.

### Statistical analyses

Demographics (age, sex, ethnicity, marital status, employment status, educational status) and clinical characteristics (pain duration, smoking status, body mass index, primary pain site, and daily morphine equivalent dose) were summarized for the three 5-HTTLPR genotype groups. The Hardy–Weinberg equilibrium (HWE) for genotypic distribution was determined using the χ^2^ test for each group [33]. Mean and standard deviation were reported for continuous variables, and count and proportion were reported for categorical variables. Group comparisons for demographic and clinical characteristics were made using unpaired *t*-tests for continuous variables and chi-square tests for categorical variables. The normal distribution of all QST measures was assessed using the Kolmogorov-Smirnov test, and the median and interquartile range (IQR) was reported. Nonparametric tests (Kruskal-Wallis test) were used to assess for the main effect of the three 5-HTTLPR genotype groups on HP perception, and individual group comparisons were performed using the Mann–Whitney U test.

Linear regression analyses were performed using HP perception as the dependent variable. Univariate regressions were performed separately on 5-HTTLPR genotype and sex, where the 5-HTTLPR high expressing category was the reference group and the intermediate and low expressing categories were the comparison groups. To test for interactions between genotype and sex, interaction terms, which were calculated as the product of the genotype group and sex, were entered in a multiple variable regression model as new variables. The final multiple variable model contained both genotype and sex as independent variables. The residual plots of all regression analyses were reviewed. The level of significance for all tests was set at *P* < 0.05, and all analyses were completed using PASW (IBM, Inc., Chicago, Il, Version 18.0).

## Results

### Sample characteristics

The study included 277 adults with chronic pain. The distribution of the high, intermediate and low expressing genotypes was 61 (22%), 149 (54%) and 67 (24%), respectively. The Hardy-Weinberg *P*-value was 0.204 which indicated no departure from equilibrium. Additional file 2: Table S1 contains a summary of demographic and clinical characteristics. Fifty-two percent (n = 143) of the cohort used daily opioids, and the mean morphine equivalent dose was 46.1 ± 77.9 mg/day. No significant differences were observed for opioid use, daily morphine equivalent dose, or pain severity.

### Heat pain perception

Additional file 3: Figure S2 contains the distribution of values for the parameters of HP perception. The values of HP 5 (Kolmogorov-Smirnov Z = 0.90, *P* = 0.386) were normally distributed, but HP 0.5 and HP 5–0.5 were not normally distributed (*P* values for Kolmogorov-Smirnov Z < 0.05).

Additional file 4: Table S2 contains the median and IQR for values of HP 0.5, HP 5, and HP 5–0.5. A significant effect of 5-HTTLPR genotype group on HP 0.5 was observed (Kruskal Wallis = 7.074, *P* = 0.029). Individual group comparisons showed that HP 0.5 was significantly greater in the intermediate expressing group compared to the high expressing group (Mann Whitney U = 3477.00, Z = -2.67, *P* = 0.009), but no significant differences were found between the intermediate and low expressing groups (*P* > 0.10) (Additional file 5: Figure S3).

In univariable linear regression analysis with HP 0.5 as the dependent variable, the intermediate expressing group was associated with significantly greater values of HP 0.5 compared to the high expressing group, and male sex was associated with significantly greater values of HP 0.5 compared to females (Additional file 6: Table S3). In a multiple variable linear regression analysis, an interaction effect between genotype and sex was investigated; however, the interaction terms were removed from the model due to lack of significance (*P* values > 0.10). In the final multiple variable regression model that included genotype and sex as independent variables, the intermediate expressing group was associated with significantly greater values of HP 0.5 compared to the high expressing group, and male sex was associated with significantly greater values of HP 0.5 compared to females (Additional file 6: Table S3).

## Discussion

The main finding of this study was that individuals in the intermediate expressing triallelic 5-HTTLPR polymorphism group had significantly greater HP thresholds compared to individuals in the high expressing group. This finding suggests that the intermediate expressing group was less sensitive to threshold-level HP stimuli compared to the high expressing group. No significant difference in HP 0.5 was observed between the intermediate and low expressing groups. In a multiple variable linear regression model that included HP 0.5 as the dependent variable and genotype and sex as independent variables, the intermediate expressing group and male sex were associated with significantly greater values of HP 0.5. However, no significant genotype-by-sex interaction effect was observed, which suggested the intermediate 5-HTTLPR genotype and sex had significant, but independent, effects on HP 0.5. Additionally, no significant differences in HP 5 or HP 5–0.5 were observed, and the demographic and clinical characteristics of the cohort were similar among the three genotype groups including sex, age, pain duration, primary pain diagnosis, opioid use, daily morphine equivalent dose, and pain severity.

Significantly greater HP thresholds among individuals in the intermediate expressing group compared to the high expressing group, but not the low expressing group, has not been reported in previous studies. In an experimental investigation that involved 44 healthy volunteers of “European descent”, subjects in the high triallelic 5-HTTLPR expressing group (n = 23) had significantly lower HP thresholds (i.e., were more sensitive to heat stimuli) compared to subjects in the low expressing group (n = 21) [12]. However, subjects from the triallelic 5-HTTLPR intermediate expressing group were not included in this particular study. In other studies conducted by the same group of investigators, no significant differences were found between the triallelic 5-HTTLPR genotype groups [10,11]. These findings were partly consistent with the observations from an experimental study that involved 58 patients with fibromyalgia and 60 healthy controls who were “Caucasian, French-speaking” residents of Quebec province, Canada [13]. In this particular study, the 5-HTTLPR polymorphism was not associated with differences in HP thresholds, but subjects were not genotyped for the rs25531 SNP which further influences the transcriptional efficiency of 5-HTT [4,5]. In a final study that involved 529 healthy subjects (72% Ashkenazi Jews of Eastern European “origin”, 20% Sephardic Jews of North African or Asian “origin”), the low expressing 5-HTTLPR genotype was associated with decreased pain inhibition compared to the high and intermediate groups [14]. Aside from differences in genotyping, variations in HP perception among previous studies, including the findings of our study, could be partly attributed to; 1) chronic pain associated neuroplastic changes in 5-HT pathways, and 2) differences in the methods used to assess thermal pain perception.

The effects of 5-HT on pain perception are varied. In the periphery, 5-HT has pro-nociceptive effects following tissue injury [34], and exogenous administration of 5-HT has been associated with hyperalgesia in preclinical and clinical studies [35-37]. At the spinal level, 5-HT transmission may either enhance descending inhibitory mechanisms or augment descending facilitatory pathways, and the balance between inhibition and facilitation may be partly influenced by neuroplastic changes that occur in response to persistent pathological pain [38]. For instance, decreases in 5-HT basal release have been observed in preclinical studies using a neuropathic pain model [39,40], but increases in 5-HT release have been reported in chronic inflammatory models of pain [41,42]. Neuroplastic changes in descending 5-HT pathways may also occur at the receptor level where alterations in 5-HT_2A_ and 5-HT_3_ receptor function have been observed in both central and peripheral tissues [43,44], and upregulation of 5-HT_1_, 5-HT_2_, 5-HT_3_ receptor subtypes have been reported in the spinal cord [44-46]. Neuroplastic changes in 5-HT pathways have also been associated with alterations in 5-HTT expression [47], which is consistent with previous studies that document a role for 5-HTT in thermal nociception [8,9]. Collectively, these studies suggest that neuroplastic changes in 5-HT pathways, in response to persistent pathological pain, could alter the effects of the 5-HTTLPR polymorphism on HP perception. Therefore, neuroplastic changes attributed to chronic pain could explain, in part, why the findings of our study, which were derived from a cohort of adults with chronic pain, differed from previous studies that predominately involved healthy volunteers.

Differences in the methods used to assess thermal pain perception could partly account for the disparate findings of previous studies. In the method of limits, a thermal stimulus is applied to the skin and increased, or decreased, at a steady rate of 1°C/s. The HP threshold is the temperature at which pain is experienced, and HP tolerance is the temperature at which the thermal stimuli can no longer be tolerated. The test is typically repeated three times, and the thermal pain threshold and tolerance are calculated by averaging the temperatures from the repeated trials. The test procedures for the method of limits have been validated [17,48], and have good reproducibility over time [49]. However, several variations in the method of limits were used in the previous studies. The Lindstedt et al., [12] study most closely adhered to previously published methodologies, and significant differences in thermal pain thresholds were observed between the high and low expressing groups. In this particular study, the heat stimulus was changed at a rate of 1.5°C/s, and three tests of HP and cold pain were performed. However, in a separate study by Lindstedt et al. [11], subjects were exposed to heat stimuli at temperatures of 46°C, 47°C, and 48°C for 15 seconds. Testing was repeated twice at each temperature, and the application of each heat stimulus was separated by a 30 second interval. In the Kosek et al. [10] study, subjects were exposed twice to a temperature of 48°C for 30 seconds, and a 10 minute interval separated each temperature exposure. The rate of rise of the heat stimuli was 0.3°C/s in the Potvin et al. study [13] where 3 tests were performed, and the rate of rise in the Treister et al. study [14] was 10°C/s where 5 tests were performed. The varying methods used to assess pain thresholds and tolerances in these studies limits the ability to draw more definitive conclusions about the effects of 5-HTTLPR polymorphism on thermal pain perception because exposure to different temperatures, or different rates of temperature change, for varying durations of time may have differential effects on the various 5-HTTLPR genotypes. However, despite these methodological variations, the effect size of the difference in thermal pain threshold between the low and high expressing 5-HTTLPR groups ranged from 0.60 to 0.32 in 3 studies that reported mean data. This range is comparable to the effect size of 0.37 for the difference in HP threshold between the intermediate and high expressing groups as observed in our current study.

In this cohort of adults with chronic pain, no significant group differences in demographic or clinical characteristics were observed based on the triallelic 5-HTTLPR genotype. This observation was similar to the findings of previous studies where no consistent differences in demographic or clinical characteristics based on 5-HTTLPR genotype have been observed in subgroups of adults with chronic pain including fibromyalgia [50-52] and chronic headache disorders [53-56]. However, various genotype-by-sex interaction effects have been reported for the 5-HTTLPR polymorphism and thermal pain perception. For example, a significant genotype-by-sex interaction effect was reported by Lindstedt et al. [12] where low 5-HTTLPR expressing females were less sensitive to cold pain, but no significant genotype-by-sex interaction was observed for HP perception. Alternatively, low expressing males had higher pain thresholds and tolerances in response to electrocutaneous stimuli in a study that involved 144 healthy volunteers of predominately “white non-Hispanic” descent who were genotyped for the 5-HTTLPR polymorphism but not the rs25531 SNP [57]. In our study, no significant genotype-by-sex interaction effect was observed; rather the intermediate expressing 5-HTTLPR genotype and male sex were independently associated with greater values of HP 0.5.

This study has limitations. The demographics and clinical characteristics of the cohort could limit the generalization of our observations. More specifically, the majority of study participants were Caucasian women residing in the United States, and only 5% were from minority populations. However, in a study that involved a random sample (N = 3,575) of community dwelling adults with chronic pain derived from the catchment area of our institution, 96% of individuals were self-identified as “white” and 56% were female [58]. Despite these demographic similarities, the clinical characteristics of patients referred for multidisciplinary pain rehabilitation at a tertiary referral medical center could be different from the clinical characteristics of a random sample of community dwelling adults with chronic pain, as previously reported [15,16]. Thus, the risk of referral bias can not be excluded, and the associations between HP perception and 5-HTTLPR polymorphisms may not be fully applicable in other populations of adults with chronic pain. Although significant differences in thermal pain perception were found among the three 5-HTTLPR genotypes in adults with chronic pain, the lack of a control group could also limit the generalization of our study findings.

## Conclusions

In this study that involved a large sample of adults with chronic pain, the three 5-HTTLPR genotypes had a significant influence on thermal pain perception. Ongoing research is warranted in order to further delineate the effects of the triallelic 5-HTTLPR genotype on pain perception in other large samples and subgroups of individuals with chronic pain.

## Competing interests

The authors state that they have no financial or non-financial competing interests.

## Authors’ contributions

WMH, WRH and JLB designed the study. WMH, WRH, JLB, HJL and DLW performed the data analysis. WMH, WRH, JLB, HJL and DLW wrote the manuscript. All authors critically revised the manuscript and approved the final form of the manuscript. All authors read and approved the final manuscript.

## Pre-publication history

The pre-publication history for this paper can be accessed here:

http://www.biomedcentral.com/1471-2350/14/78/prepub

## Supplementary Material

Additional file 1: Figure S1 The patient grades the intensity of the stimulus from 0 to 10 where 0 indicates no pain. Click here for file

Additional file 2: Table S1. Demographic and clinical characteristics of study participants. Click here for file

Additional file 3: Figure S2 Distribution of QST values for HP 0.5 **(A)**, HP 5 **(B) **and HP 5-0.5 **(C)** expressed in units of just noticeable difference (JND). Click here for file

Additional file 4: Table S2 Median values and interquartile range (IQR) of heat pain (HP) perception for the triallelic 5-HTTLPR Polymorphism. Click here for file

Additional file 5: Figure S3 Median value of HP 0.5 and 95% confidence interval for the 5-HTTLPR genotype groups in units of just noticeable difference (JND). Click here for file

Additional file 6: Table S3 Linear regression analyses with HP 0.5 as the dependent variable. Click here for file
