# Supplementary figures and images for: Associations between serotonin transporter gene polymorphisms and heat pain perception in adults with chronic pain
Source: BMC Med Genet. 2013 Jul 30;14:78. doi: 10.1186/1471-2350-14-78 (PMC3737051; doi:10.1186/1471-2350-14-78)

## Slide 1
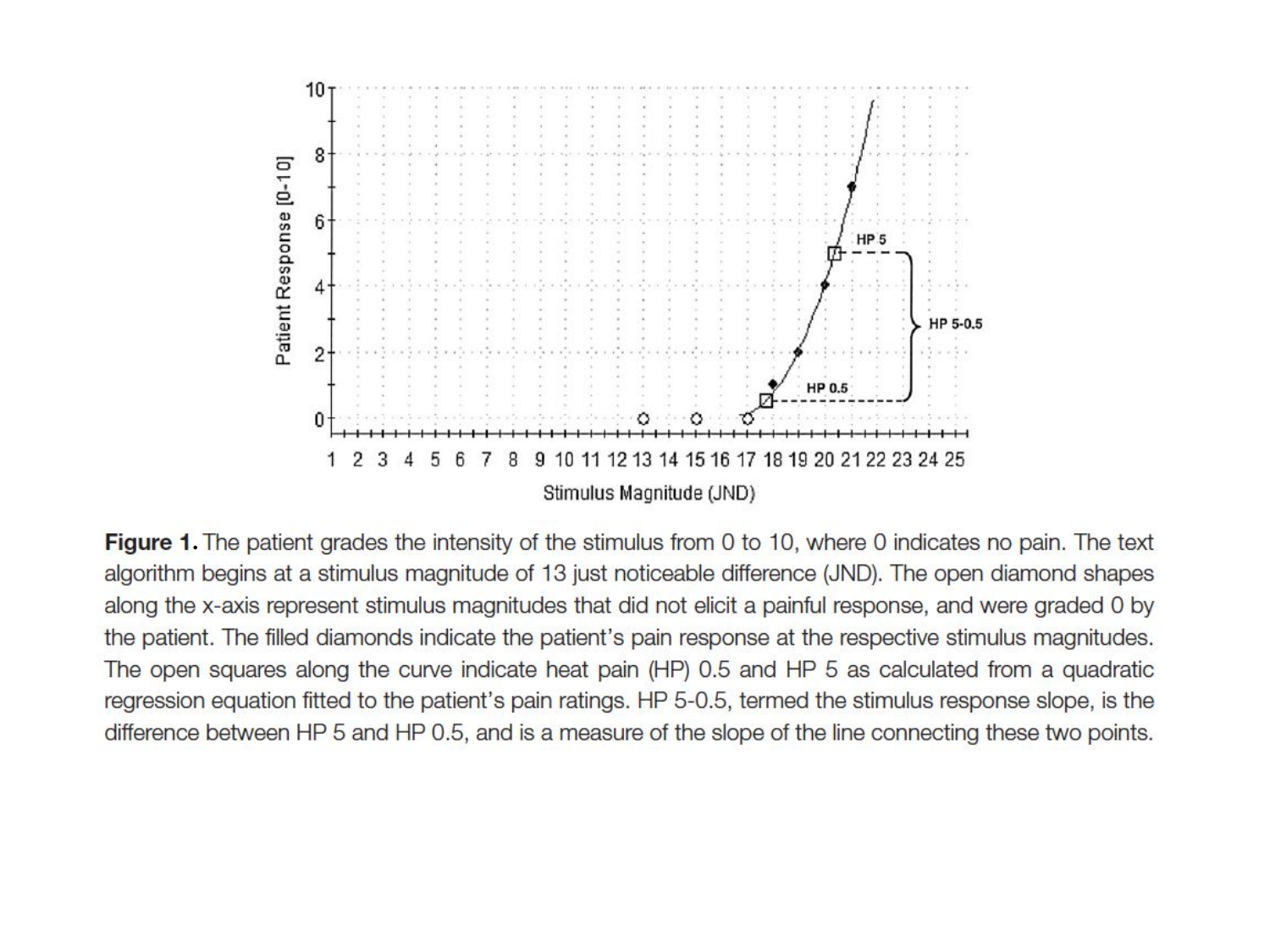

.

Supplement: Additional file 1: Figure S1 — The patient grades the intensity of the stimulus from 0 to 10 where 0 indicates no pain. [file 1471-2350-14-78-S1.pptx]
