# Supplementary material for: Associations between serotonin transporter gene polymorphisms and heat pain perception in adults with chronic pain
Source: BMC Med Genet. 2013 Jul 30;14:78. doi: 10.1186/1471-2350-14-78 (PMC3737051; doi:10.1186/1471-2350-14-78)
Supplement: Additional file 2: Table S1. — Demographic and clinical characteristics of study participants. [file 1471-2350-14-78-S2.pdf]

Table 1. Demographic and clinical characteristics of study participants.

Characteristic	Total (n = 277)	5-HTTLPR Genotype<br>High Expressing (n = 61)	Intermediate (n = 149)	Low Expressing (n = 67)
Age, years, *mean* ± *SD*	45.5 ± 13.2	46.7 ± 12.7	45.6 ± 13.3	44.3 ± 13.4
Sex, *No. female (%)*	185 (67)	44 (72)	98 (66)	43 (64)
Ethnicity				
Caucasian *No. (%)*	263 (95)	57 (93)	145 (97)	61 (91)
African American	2 (< 1)	0	2 (1)	0
Asian	2 (< 1)	0	0	2 (3)
AI/AN	3 (1)	1 (2)	2 (1)	0
Pacific Islander	2 (<1)	0	0	2 (3)
Hispanic	5 (2)	3 (5)	0	2 (3)
Marital status, *No. married (%)*	175 (63)	38 (62)	95 (64)	42 (63)
Education, years, *mean* ± *SD*	15.0 ± 2.9	14.6 ± 2.7	15.3 ± 3.0	14.8 ± 2.7
Pain duration, years, *mean* ± *SD*	9.3 ± 8.3	8.4 ± 8.9	9.7 ± 7.9	9.2 ± 8.6
BMI (kg/m^2), *mean* ± *SD*	29.2 ± 6.9	28.9 ± 8.0	29.3 ± 6.3	29.4 ± 7.3
Current employment, *yes (%)*	85 (31)	16 (26)	48 (32)	21 (31)
Primary pain diagnosis				
Low back pain, *No. (%)*	62 (22)	9 (15)	32 (21)	21 (31)
Fibromyalgia	60 (23)	13 (21)	32 (21)	15 (22)
Abdominal	21 (8)	9 (15)	8 (5)	4 (6)
Generalized	37 (13)	7 (11)	23 (15)	7 (10)
Chronic headache	28 (10)	4 (7)	18 (12)	6 (9)
Pelvic	10 (4)	2 (3)	7 (5)	1 (2)
Lower extremity	15 (5)	4 (6)	7 (5)	4 (6)
Facial	5 (2)	1 (2)	3 (2)	1 (2)
Neck	21 (7)	8 (13)	11 (7)	2 (3)
Upper extremity	13 (5)	3 (5)	7 (5)	3 (4)
Chest wall	3 (1)	0	1 (<1)	2 (3)
Other	2 (< 1)	1 (2)	0	1 (2)
Opioid use, *yes (%)*	143 (52)	28 (46)	80 (54)	35 (52)
Morphine equivalent dose (mg/day), *mean* ± *SD*	46.1 ± 77.9	43.3 ± 82.2	50.9 ± 85.6	38.1 ± 51.5
Smoking status, *yes (%)*	51 (18)	12 (20)	25 (17)	14 (21)
Pain severity*	51.0 ± 7.1	50.3 ± 7.4	51.0 ± 7.1	51.4 ± 6.7

*pain severity subscale of the Multidimensional Pain Inventory.
