# Supplementary material for: Associations between serotonin transporter gene polymorphisms and heat pain perception in adults with chronic pain
Source: BMC Med Genet. 2013 Jul 30;14:78. doi: 10.1186/1471-2350-14-78 (PMC3737051; doi:10.1186/1471-2350-14-78)
Supplement: Additional file 3: Figure S2 — Distribution of QST values for HP 0.5 (A), HP 5 (B) and HP 5-0.5 (C) expressed in units of just noticeable difference (JND). [file 1471-2350-14-78-S3.pptx]

## Slide 1
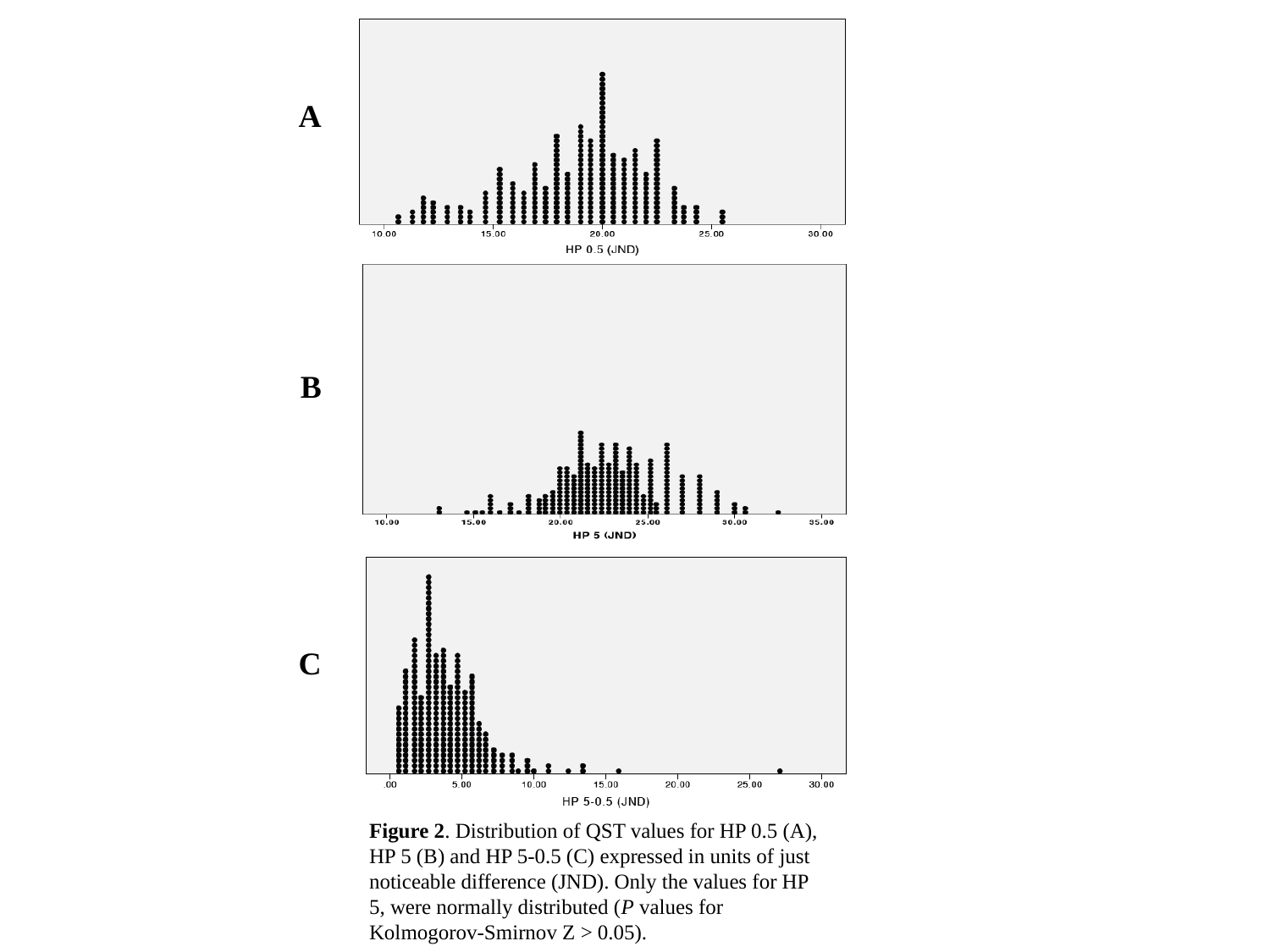

A
B
C
Figure 2. Distribution of QST values for HP 0.5 (A), HP 5 (B) and HP 5-0.5 (C) expressed in units of just noticeable difference (JND). Only the values for HP 5, were normally distributed (P values for Kolmogorov-Smirnov Z > 0.05).
