# Supplementary material for: Associations between serotonin transporter gene polymorphisms and heat pain perception in adults with chronic pain
Source: BMC Med Genet. 2013 Jul 30;14:78. doi: 10.1186/1471-2350-14-78 (PMC3737051; doi:10.1186/1471-2350-14-78)
Supplement: Additional file 4: Table S2 — Median values and interquartile range (IQR) of heat pain (HP) perception for the triallelic 5-HTTLPR Polymorphism. [file 1471-2350-14-78-S4.docx]

| Table 2. Median values and interquartile range (IQR) of heat pain (HP) perception for the triallelic 5-HTTLPR  polymorphism. | | | | |
| --- | --- | --- | --- | --- |
| HP Parameter | Total  (n = 277) | High Expressing  (n = 61) | Intermediate  (n = 149) | Low Expressing  (n = 67) |
| HP 0.5, *median* *(IQR)* |  |  |  |  |
| JND* | 19.40 (17.05, 21.05) | 18.20 (16.60, 20.10) | 20.00 (18.00, 21.10) | 18.90 (15.30, 21.60) |
|  |  |  |  |  |
| HP 5 |  |  |  |  |
| JND | 22.80 (20.95, 24.75) | 22.20 (20.75, 24.00) | 22.90 (21.00, 25.00) | 23.00 (20.40, 25.50) |
|  |  |  |  |  |
| HP 5-0.5 |  |  |  |  |
| JND | 3.50 (2.15, 5.20) | 3.50 (2.50, 5.30) | 3.20 (1.95, 4.95) | 3.9 (2.70, 5.50) |
|  |  |  |  |  |

*****just noticeable difference
