# Supplementary material for: Associations between serotonin transporter gene polymorphisms and heat pain perception in adults with chronic pain
Source: BMC Med Genet. 2013 Jul 30;14:78. doi: 10.1186/1471-2350-14-78 (PMC3737051; doi:10.1186/1471-2350-14-78)
Supplement: Additional file 5: Figure S3 — Median value of HP 0.5 and 95% confidence interval for the 5-HTTLPR genotype groups in units of just noticeable difference (JND). [file 1471-2350-14-78-S5.pptx]

## Slide 1
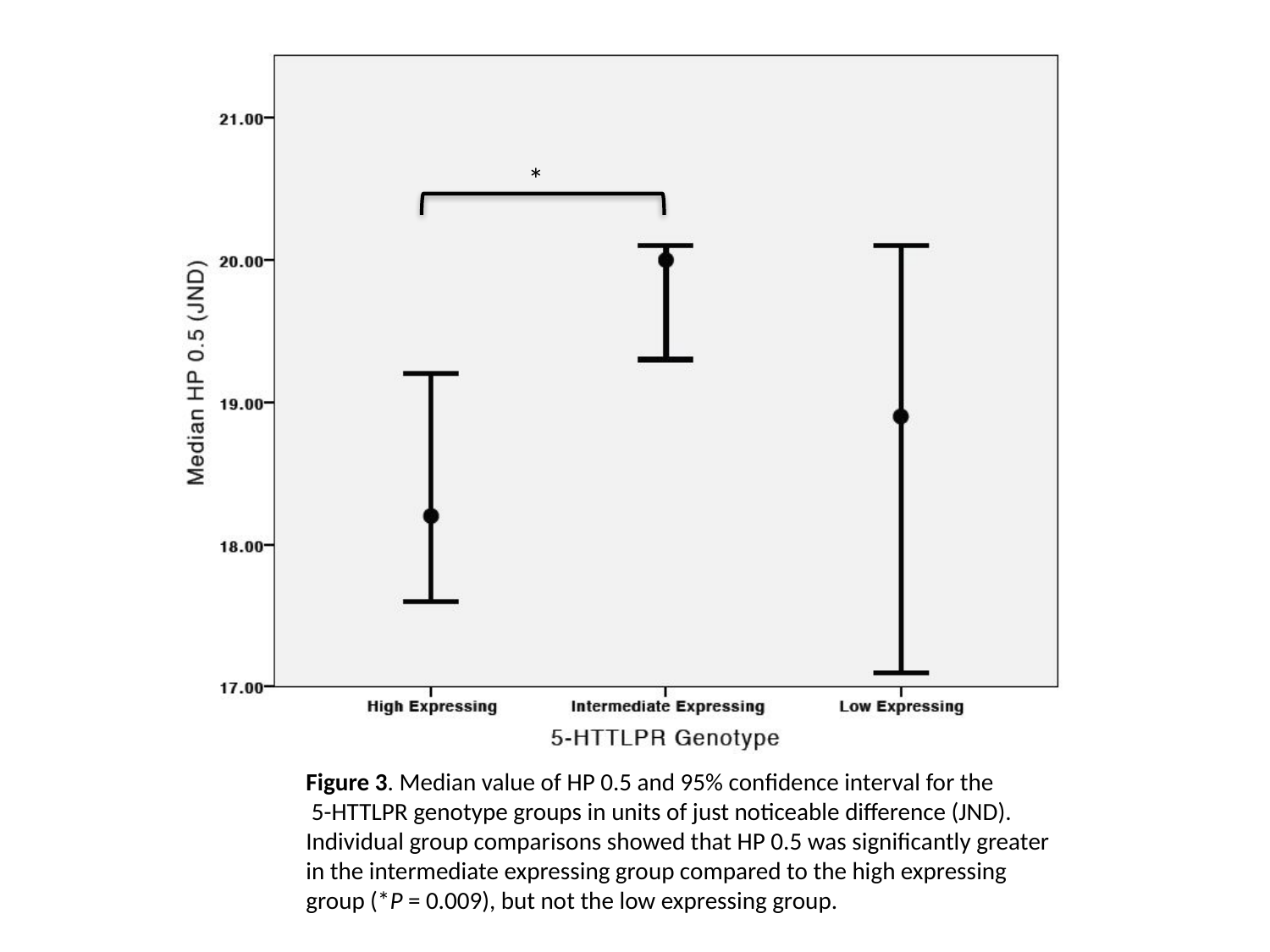

*
Figure 3. Median value of HP 0.5 and 95% confidence interval for the
 5-HTTLPR genotype groups in units of just noticeable difference (JND).
Individual group comparisons showed that HP 0.5 was significantly greater
in the intermediate expressing group compared to the high expressing
group (*P = 0.009), but not the low expressing group.
