# Supplementary material for: Associations between serotonin transporter gene polymorphisms and heat pain perception in adults with chronic pain
Source: BMC Med Genet. 2013 Jul 30;14:78. doi: 10.1186/1471-2350-14-78 (PMC3737051; doi:10.1186/1471-2350-14-78)
Supplement: Additional file 6: Table S3 — Linear regression analyses with HP 0.5 as the dependent variable. [file 1471-2350-14-78-S6.docx]

| Table 3. Linear regression analyses with HP 0.5 as the dependent variable. | | | | |
| --- | --- | --- | --- | --- |
|  | | | | |
| Independent variables | B coefficient univariate analysis (95% CI) | *P* value | B coefficient multiple variable analysis* (95% CI) | *P* value |
|  |  |  |  |  |
| 5-HTTLPR genotype |  |  |  |  |
| High expressing | 0.000 |  | 0.000 |  |
| Intermediate | 1.007 (0.265, 1.749) | 0.008 | 1.011 (0.079, 1.942) | 0.034 |
| Low expressing | -0.640 (-1.512, 0.232) | 0.149 | 0.045 (-1.040, 1.130) | 0.935 |
| Male sex | 0.947 (-0.159, -1.735) | 0.019 | 0.921 (0.138, 1.703) | 0.021 |

* Adjusted for all other factors listed in the table.
